# Rapid Antigen Test Combine with Nucleic Acid Detection: A Better Strategy for COVID-19 Screening at Points of Entry

**DOI:** 10.1007/s44197-021-00030-4

**Published:** 2022-01-03

**Authors:** Zhiqing Zhan, Jie Li, Zhangkai J. Cheng

**Affiliations:** 1 Department of Allergy and Clinical Immunology, Guangzhou Institute of Respiratory Health, State Key Laboratory of Respiratory Disease, National Clinical Research Center of Respiratory Disease, First Affiliated Hospital of Guangzhou Medical University, Guangzhou, China; 2 Guangzhou Medical University, Guangzhou, China

**Keywords:** COVID-19, Rapid antigen test, Delta variant

## Abstract

The coronavirus disease 2019 (COVID-19) pandemic has imposed an enormous disease burden worldwide, and the Delta variant now has become dominant in 53 countries. Recently published studies have shown that during periods of high viral load, rapid antigen tests (RAT) yield similar results to reverse transcriptase-polymerase chain reaction (RT-PCR) tests, and when used in serial screening (e.g., every three days), it has a high sensitivity. In this perspective, we recommend RT-PCR combined with RAT at points of entry: (i) RAT can be added to the detection phase at ports of entry to detect asymptomatic infections as early as possible; (ii) RAT can be added to post-entry quarantine every three days or less to reduce the rate of missed detection in later quarantine; (iii) Adding regular RAT to regular PCR testing for key airport personnel to prevent cross-infection and conduct closed-off management. In the face of sporadic Delta variant outbreaks, the combination of the two could help rapid triage and management of suspected populations at an early stage and thus contain the outbreak more quickly and effectively. We also discuss the issue whether the current antigen detection reagents can cope with various SARS-CoV-2 variants.

## Introduction

On July 21, 2021, a small superspreader event occurred at Lukou International Airport in Nanjing, China. During a routine nucleic acid amplification test (NAAT) conducted on airport staff, nine returned positive results (seven confirmed cases, two asymptomatic), all of which were infected with the Delta variant. In the days that followed, the chain of transmission continued to extend, forming a chain of intra-city and inter-provincial transmission, linking twenty-two cities in eight provinces in China. As of August 2, 327 confirmed cases had been reported to be from Nanjing Lukou International Airport or its associated cases, of which 50% were from centralized isolation sites. In the context of emerging severe acute respiratory syndrome coronavirus 2 (SARS-CoV-2) variants, increasing the number of tests during the quarantine period, shortening the regular testing period for staff with a high risk of exposure at airports, and identifying COVID-19 patients as early as possible through rapid screening may aid the control of the epidemic at border entry. The current standard for detection of SARS-CoV-2 is NAAT such as reverse transcriptase polymerase chain reaction (RT-PCR) [1]. RT-PCR tests have high accuracy, but it has high requirements for testing equipment and operators, and is expensive and time-consuming (most would take 1–3 days). During the waiting time for the test results, infections can spread, and this is particularly troublesome for border entry management. In this case, it is desirable to shorten the time between testing and confirmation of results. However, it is difficult for RT-PCR to implement this requirement with its longer turnaround time for lab-based tests.

On the other hand, rapid antigen tests (RAT) most only take 15–30 min to obtain test results and are simple and inexpensive [1]. Unlike RT-PCR, the antigens need not be multiplied to sufficient levels for the test to detect the virus. These tests act on the available load of the virus in the sample. If the viral load in the sample is low, the test can be negative. The pooled sensitivity and pooled specificity of Standard Q COVID-19 Ag test, a RAT in the emergency use listing that was recommended by the WHO, were 0.83 (95% CI 0.63–0.94) and 0.99 (95%CI, 0.95–1.00), respectively [1]. Previous studies showed that STANDARD F COVID-19 Ag FIA is highly specific (98.4%, 95% CI 96.0–99.6%) for SARS–CoV-2 Ag detection in NPS, and highly sensitive (95.2%, 95% CI 76.2–99.9%) for samples with cycle threshold (Ct) value lower than 25 [2–4]. A systematic review and meta-analysis reported that in Ct-value ≤ 25 and ≤ 30, the pooled sensitivity of RATs was 0.94 (95% CI 0.84–0.98) and 0.84 (95% CI 0.77–0.93), respectively, compared with RT-PCR, indicating that RAT may help to identify individuals with high viral load and thus minimizing forward transmission (given timely results reporting) [1].

**Fig.1:**
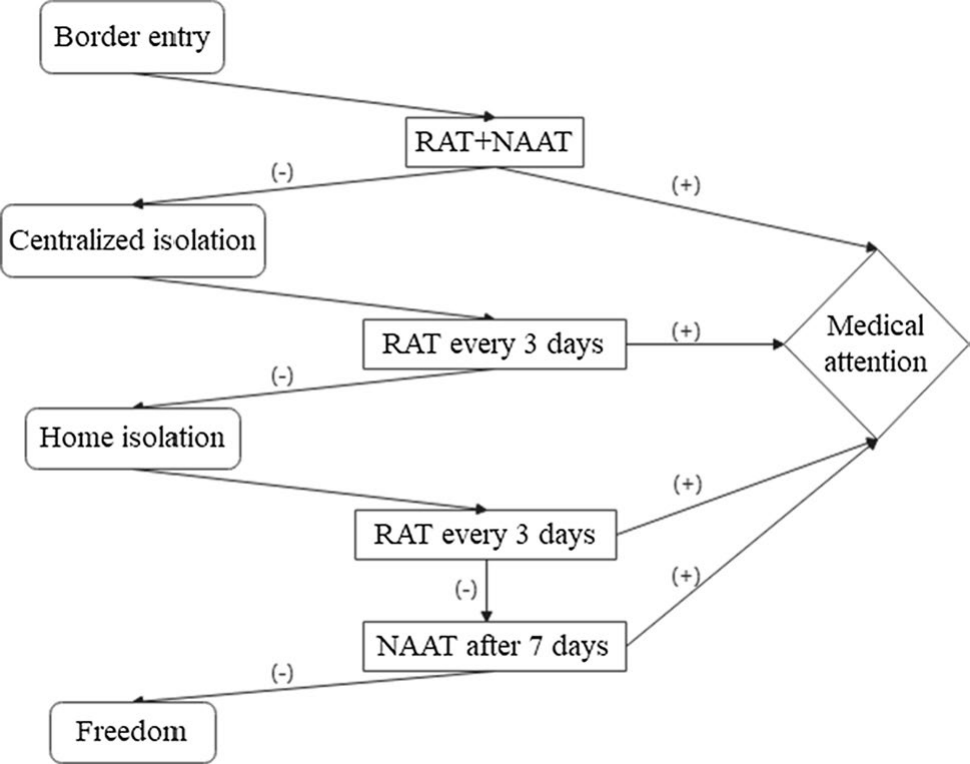
The recommended detection process at Points of Entry. ( +): the results of detection are positive, (−): the results of detection are negative, *RAT + NAAT* Rapid Antigen Test combined with nucleic acid detection

The Delta variant now has become dominant in 53 countries. According to a study in China, people infected with Delta had viral loads (Ct = 24.00, IQR = 19.00–29.00) up to 1260 times higher than those in people infected with the original strain, which would boost the sensitivity of RAT [5]. A study reported that when used in serial screening (i.e., at least every 3 days), Quidel SARS Sofia antigen fluorescent immunoassay (FIA) has a sensitivity of > 98% for identifying infected individuals, which is not significantly different from RT-PCR tests, and may help compensate for the limited sensitivity of detecting early infection [6]. We recommend the current detection strategy at points of entry to be optimized as follows (Fig. 1): (i) The current practice for detection (RT-PCR tests at the port of entry) takes 1–3 days for the results to return. We suggest combining RAT with RT-PCR tests at border entry. Subjects with positive results for either test should be listed as key screening targets and quarantined separately. This new strategy may help identify asymptomatic infections in 15–30 min (i.e., time taken for RAT results to return) and cut off further viral spread. (ii) RAT can be added to post-entry quarantine every 3 days or less to reduce the rate of missed detection in later quarantine; (iii) Add regular RAT to regular RT-PCR testing for key airport personnel to prevent cross-infection and conduct closed-off management. In the face of sporadic Delta variant outbreaks, the combination of the two could help to contain the outbreak more quickly and effectively.

Faced with mutations, a concern is: can the current antigen detection reagents cope with various SARS-CoV-2 variants? So far, Bourassa et al. [7] reported an approximate 1000-fold loss in sensitivity for the Sofia SARS Antigen FIA test associated with the D399N mutation and Jian et al. [8] found that the variants undetected by the Panbio COVID-19 RAT may be due to the T135I mutation in the N protein. D399N mutation is rare, appearing in only 0.02% of genomes worldwide at the time of writing. Whether mutations in these strains affect the accuracy of antigen detection depends on whether the N or S protein of the virus can be detected. Mutations in the S protein are the most common, but fortunately few antigen tests are specific to this biomarker, and most test reagents are specific to the N protein or both N and S protein. Although mutations at the N-terminal of the N-antigen have been reported, most current antigen assays target the C-terminal and therefore should not affect antigen detection performance in theory. We suggest concomitant paired RAT and molecular diagnostic methods to detect SARS-CoV-2. False-negative results could be rapidly corrected using confirmatory RT-PCR results. Confirmation by laboratory testing of clinical samples representing each variant is also recommended.

## Conclusion

Considering that RAT is faster than RT-PCR, and they yield similar results during periods of high viral load, we believe that the use of RT-PCR combined with RAT at border entry can better identify individuals with contagious levels of viral load, which is also of great help at points of entry for rapid triage and management of suspected populations at an early stage.
